# Ashwagandha: Is It Safe? Part 1: A Regulatory Review

**DOI:** 10.1002/ptr.70151

**Published:** 2025-12-19

**Authors:** T. Brendler, R. Al‐Mondhiry, L. Lang, R. Marles, M. Tallon, A. Raghu

**Affiliations:** ^1^ Department of Botany and Plant Biotechnology University of Johannesburg Johannesburg South Africa; ^2^ Pharmacognosy Institute, School of Pharmacy, University of Illinois Chicago Chicago Illinois USA; ^3^ Amin Wasserman Gurnani LLP Washington District of Columbia USA; ^4^ Complementary Medicines Australia Mawson Australian Capital Territory Australia; ^5^ Health Products and Food Branch, Health Canada Ottawa Ontario Canada; ^6^ Legal Foods Ltd. Ely Cambridgeshire UK; ^7^ Directorate General of Health Services New Delhi India

**Keywords:** adaptogen, ashwagandha, regulations, safety, *Withania*

## Abstract

Over the last decade, ashwagandha ( *Withania somnifera* (L.) Dunal, AS) has been brought under increasing scrutiny by EU regulators regarding its safety for the use in food supplements, culminating in a recent recommendation for an Article 8 procedure according to Regulation (EC) No. 1925/2006 in the European Union (EU). Once executed, this could lead to a ban on its use as an ingredient in food supplements. This review summarizes how AS is regulated in some of the primary world marketplaces.

## 1 Introduction

Ashwagandha ( *Withania somnifera* (L.) Dunal), also known as winter cherry, is a prominent herb used in the traditional medicines of India represented by Ayurveda, Yoga & Naturopathy, Unani, Siddha, Sowa‐Rigpa and Homeopathy (Ayush). Numerous Ayurvedic texts and treatises highlight its diverse health benefits, particularly its rejuvenating effects and capacity to augment the human body's response to stress. In Ayurveda, AS root is valued as a “rasayana” (rejuvenating tonic), supporting weight gain, vitality and aphrodisiac effects (Balasubramani et al. 2011). In addition to its extensive use in Ayurveda, AS is incorporated into other Ayush systems such as Siddha, Unani, Sowa Rigpa and homoeopathy, attesting to its therapeutic versatility and cultural significance across traditional medicinal paradigms.

In the European Union (EU), AS is regulated as a food supplement (EU Directorate‐General for Health and Food Safety 2024). Despite its long‐standing traditional medicinal use in Ayush, EU regulators initially questioned the safety of AS in 2013 (Klenow et al. 2013).

In December 2023, VigiBase, the World Health Organization's (WHO) global database of individual case safety reports of suspected adverse drug reactions, maintained by the Uppsala Monitoring Centre on behalf of WHO, contained 15 reports of hepatobiliary disorders and investigations associated with AS (Lim and Barnes 2024). Additionally, adverse event reports held by national competent authorities—such as the Australian Therapeutic Goods Administration (TGA, see below), which published a safety advisory in February 2024 (Therapeutic Goods Administration 2024)—were primarily related to digestive complaints.

These concerns led to a recent recommendation by the Heads of Food Safety Agencies (HoA) working group on food supplements for an Article 8 procedure according to Regulation (EC) No 1925/2006 (Heads of Food Safety Agencies 2024). If implemented, this may lead to a ban on the use of AS as an ingredient of food supplements. Since AS is not considered a traditional herbal medicine in the EU (HMPC 2013), its ban in the food supplement category may result in the removal of the ingredient from the EU marketplace entirely.

Different markets regulate AS differently, the majority as supplements and/or foods. Responses of competent authorities to AS‐related safety concerns have varied widely, from outright bans from the supplement category to observation and interpretation of the available data. In the following we showcase the status of AS in regulatory systems around the world. This review is intended to inform regulators, healthcare practitioners and consumers on the regulatory status of AS in key marketplaces as well as to provide recommendations for harmonized regulatory interventions.

## 2 Methods

Markets were chosen based on tradition, use and volume of consumption. Both in the EU and North America, AS products are top performers in the supplement category (Smith et al. 2024). The UK population includes a significant diaspora of traditional users. Traditional use is rooted in India's medicinal paradigms. Another inclusion criterium was whether and how national competent authorities have collected adverse event reports and initiated interventions as a consequence.

## 3 Results

### 3.1 Ashwagandha in India

In India, Ayush traditional medicine systems are widely practiced and documented. The first exclusive all‐India survey on Ayush conducted by the National Sample Survey Office (NSSO), Ministry of Statistics and Program Implementation, Govt. of India (July, 2022 to June, 2023) as a part of the 79th round of the National Sample Survey (NSS) revealed that 94.8% of people in rural areas and 96% of people in urban areas are aware of the Ayush systems of medicine (Government of India 2023).

National regulations for traditional medicines such as Ayush have been established under the Drugs & Cosmetics Act, 1940 and rules thereunder (Government of India 2016a), which documented the provisions for ensuring the safety and efficacy of Ayush drugs including AS, as established in authoritative texts specified in the Act's First Schedule.

Ayush drugs are defined in Section 3(a) of the Act as medications intended for diagnosis, treatment, mitigation or prevention of diseases in humans or animals, manufactured per the formulae in the authoritative texts of Ayush systems listed in the First Schedule. Patent or Proprietary Medicines are defined under Section 3(h) as those formulations that contain ingredients listed in the authoritative texts of these systems, with restrictions on parenteral administration and certain proprietary formulations.

The Drugs & Cosmetics Act and rules thereunder prescribe the legal requirements for the manufacture for sale of medicines in Ayush systems, among others as it relates to regulating the quality, safety and efficacy of the medicines. The Drugs & Cosmetics Act and rules thereunder establish very specific provisions regarding quality, standards, labeling and manufacturing. They also provide for the constitution of an Ayurvedic, Siddha and Unani Consultative Committee (ASUDCC) and Ayurvedic, Siddha and Unani Technical Advisory Board (ASUDTAB) to advise the government in the technical matters of Ayurveda, Siddha and Unani drugs and their administration throughout the country.

The Act also defines misbranded, adulterated and spurious drugs. Manufacture, sale and distribution of any Ayush drug against the license conditions and standards is prohibited. Under the Drugs & Cosmetics Act, the central government has the power to prohibit in the public interest the manufacture, sale, etc., of any medicine, which does not have the therapeutic value claimed. Furthermore, the Act empowers the central and state governments to appoint inspectors for Ayush drugs. Penal provisions for manufacture for sale or distribution of Ayush drugs in contravention of the provisions of the Act have also been prescribed.

The Act mandates that state‐level drug controllers or licensing authorities enforce quality control and regulatory standards for Ayush drugs, ensuring adherence to safety and efficacy requirements. Schedule T of the Drugs & Cosmetics Act outlines good manufacturing practices (GMPs) for Ayush medicines, specifying factory requirements, recommended machinery and equipment, and guidelines for in‐house quality control. A list of poisonous substances under Ayush systems is detailed in Schedule E (1) of the Drugs Rules, 1945. Products containing these substances must be labeled with a warning in English and Hindi, though AS is not included in this list.

Foods and food supplements claiming health benefits are a major and fast‐growing food category in India. In 2016, the Food Safety and Standards Authority of India (FSSAI) inter‐alia laid down the standards for health supplements containing AS (Government of India 2016b).

The Pharmacopoeia Commission for Indian Medicine and Homoeopathy (PCIM&H), under the Ministry of Ayush, sets pharmacopeial standards for identity, purity and strength for Ayush drugs. Standards for identity, purity and strength of AS have been included in respective pharmacopoeia of Ayush. The Ministry also supports WHO‐compliant certification programs and quality certification schemes, such as the WHO‐COPP, for Ayush products.

The Bureau of Indian Standards (BIS) is the National Standards Body of India, responsible for the development of Indian standards for products, processes and services related to all consumer goods. BIS formulates standards in line with the national priorities for various sectors. BIS has created a dedicated department of standardization in the Ayush systems. A total 91 Ayush related standards including 80 standards for single herbs such as AS have been published by BIS to date (Bureau of Indian Standards 2022).

The National Pharmacovigilance Program for Ayush drugs is coordinated by the All‐India Institute of Ayurveda (AIIA) and involves a three‐tier structure for monitoring and reporting adverse drug reactions and misleading advertisements to respective state authorities.

### 3.2 Ashwagandha in the European Union

In 2013, the German Federal Institute for Risk Assessment (BfR) published a level A risk assessment (Klenow et al. 2013) (conducted between 2010 and 2012) of the use of certain botanicals and preparations in food (supplements)—among them AS—in accordance with European Food Safety Authority (EFSA) guidance (EFSA Scientific Committee 2009). The report recommended inclusion of AS in list C of Appendix III of Directive EC 1925/2006 (European Union 2006b) as amended (Article 8 procedure), thus effectively prohibiting it from use in food (supplements). This recommendation was based on multiple safety concerns, such as the possibility of undesirable endocrinological effects, the traditionally documented use as an abortifacient, the absence of toxicological and safety data and the lack of data on safe dosing and exposure. Consequently, EFSA's presumption of safety paradigm was deemed inapplicable.

According to a co‐author of the BfR recommendations, these were not enacted by EU regulators at the time because there was no consensus on whether and how Article 8 procedures should be conducted among EU member states. As a result, and with Germany as driving force, a working group of the HoA was created in 2019 (active since 2020) to systematically advance Article 8 procedures (pers. comm. Klaus Peter Latté, July 2024).

In parallel, in 2013, the Committee on Herbal Medicinal Products (HMPC) of the European Medicines Agency (EMA), concluded that requirements of Article 1 and 16a(1)(d) of Directive 2001/83/EC (European Union 2001) were not fulfilled, and, consequently, a community herbal monograph on AS could not be established at that time (HMPC 2013).

By 2013, EFSA's assessment of “voluntary statement[s] that refers to the relationship between food and health” (i.e., health claims) according to Article 13.1 of Regulation EC 1924/2006 (European Union 2006a) (Nutrition and Health Claim Regulation, NHCR) had left multiple health claims pertaining to AS supplements on hold. “On hold” claims may be used while they are still under consideration, subject to the transition measures in Article (28)(5) of the NHCR. The proposed wording can be found in EFSA's “on‐hold” list (EFSA 2021).

Consequently, supplement products currently in the EU (and UK, see below) markets are utilizing this pool of “available” claim language to the full extent. A recent implementation report and resulting resolution of the European Parliament (European Union 2024) intends to push the European Council and Commission into acting on the “on‐hold” list, associated incongruities and the lack of harmonization between member states. This may give HoA's 2024 recommendations (see below) extra traction.

In 2019 (published in 2020), on behalf of the Danish Food and Drug Administration, the DTU Food Institute conducted a risk assessment of AS (DTU Fødevareinstituttet 2020). Once again, significant safety concerns were derived from the purported traditional use as an abortifacient, its potential effect on sex and thyroid hormone levels, its potential inhibitory effect on acetylcholinesterase, its potential effect on the immune system and five cases of liver damage associated with the consumption of AS. The report further concluded that, based on the available data, no safe dose of intake can be determined.

Per resolution 7/2020 (Główny Inspektorat Sanitarny 2021), Polish competent authorities, citing the DTU report, limited daily intake to 3 g of powdered root, with no more than 10 mg withanolides daily.

In 2021, the French Agency for Food, Environmental and Occupational Health & Safety (ANSES) initiated an appraisal of the risks associated with the use of AS preparations in food supplements (ANSES 2021).

In September 2022, the Swedish Food Agency announced that the Danish risk assessment can also be applied in Sweden but that the municipalities may decide on a case‐by‐case basis (Livsmedelsverket 2022).

Based on the DTU assessment, Denmark banned the use of AS in food (supplements) in 2023 (Fødevarestyrelsen 2024).

In March 2024, the Dutch National Institute for Public Health and the Environment (RIVM) published a risk assessment of AS, finding that AS products currently in the Dutch marketplace “may lead to adverse effects such as liver injury, thyrotoxicosis and suppression of the adrenal system in sensitive people. It is unknown which individuals are sensitive to *Withania somnifera* . In addition, according to the WHO, *W. somnifera* was historically used to induce abortions, indicating that *W. somnifera* could be harmful to the unborn child. No suitable reproduction toxicity studies were available to exclude that herbal preparations containing *W. somnifera* can cause this effect. For *W. somnifera* tea, no toxicological data are available, and the information on the exposure is limited. In the absence of sufficient data, the conclusion for food supplements is assumed to also apply to *W. somnifera* tea. As a precaution, RIVM advises consumers, and in particular pregnant women, to not use herbal preparations containing *W. somnifera* ” (Rijksinstituut voor Volksgezondheid en Milieu 2024). This ruling was updated in 2025 in a proposed amendment to the Commodities Act on Food Supplements and Herbal Preparations,^1^ which if enacted would ban the use of AS in food supplements.

This was followed by publication of a risk assessment conducted by the French Agency for Food, Environmental and Occupational Health & Safety in April 2024 (ANSES 2024) that advised consumers with thyroid, hepatic or cardiac pathologies or hyperandrogenism, pregnant and breastfeeding women, people taking sedatives and children up to the age of 18 to avoid consuming such dietary supplements.

In June 2024, HoA published a first report of its food supplements working group, in which AS was recommended to the European Commission for a prioritized Article 8 procedure, based on “(possible) carcinogenic, mutagenic or reprotoxic properties”, citing the concerns of the DTU report (effects on reproduction, thyroid hormones, acetylcholinesterase, the immune system and liver toxicity) (Heads of Food Safety Agencies 2024). According to the October newsletter of the International Alliance of Dietary/Food Supplement Associations (IADSA), “the Commission has requested that Member States provide detailed information on how these substances are currently regulated, whether national risk assessments have been conducted, and if there are concerns related to their safety or overconsumption. The Commission is particularly interested in whether these substances are classified as food or medicinal products in different Member States. This data will help an assessment of whether existing risk management measures are sufficient or if further actions are required” (IADSA 2024), which implies that the initiation of an Article 8 Procedure is not imminent until such data has been received and evaluated by the EC.

The German BfR in its Communication 39 of September 10, 2024, reiterated notions of paucity of safety data and aforementioned safety concerns for a conclusive risk assessment or the establishment of safe intake levels. Referencing its 2012 risk assessment, and backed by DTU, RIVM, ANSES and TGA (see above and below), it invoked the cautionary principle and recommended that “if the possibility of harmful effects on health has been identified but scientific uncertainty persists, the substance shall be placed in Annex III, Part C (Substances under Community scrutiny)” of Regulation EC 1925/2006. In the meantime, “especially children, pregnant women, breastfeeding mothers and people with liver disease should avoid” AS food supplements, and the general population is called upon to exercise restraint (BfR 2024).

Until further harmonization, member states apply national rule as a governing principle in apparent supersession of mutual recognition stipulations of Regulation EU 2019/515 and have developed national positive and/or negative lists for the use of botanicals in food supplements. Table 1 summarizes the regulatory status of such lists, and Table 2 provides summaries of member state specific stipulations pertaining to AS.

**TABLE 1:** EU national legislation for the use of botanicals in food supplements (European Commission 2020).

Member state	Positive list	Negative list	Status
Austria	Y	Y	Official guideline
Belgium	Y	Y	Legally binding
Bulgaria	N	Y	Legally binding
Croatia	Y	Y	Legally binding
Czechia	Y	Y	Legally binding
Denmark	N	Y	Official guideline
Estonia	N	Y	Official guideline
Finland	N	Y	Official guideline
France	Y	N	Legally binding (BELFRIT list for mutual recognition)
Germany	Y	Y	Official guideline
Hungary	N	Y	Legally binding
Ireland	N	Y	Official guideline
Italy	Y	N	Legally binding
Latvia	N	Y	Legally binding
Lithuania	N	Y	Legally binding
Netherlands	N	Y	Legally binding
Poland	Y	N	Unofficial internal guideline
Romania	Y	Y	Negative list—legally binding Positive list—unofficial guideline
Slovenia	Y	Y	Official guideline
Sweden	N	Y	Official guideline

**TABLE 2:** European national regulations governing the use of AS in food supplements (partially overruled by recent regulatory intervention, see above).

Country	Regulatory status	Reference
Austria	Harmonized with *Stoffliste* (see Germany, not clear if overruled).	https://www.lebensmittelbuch.at/beschluesse/leitlinien‐richtlinien‐empfehlungen‐usw‐der‐codexkommission/nahrungsergaenzungsmittel/empfehlung‐fuer‐die‐verwendung‐von‐pflanzen‐und‐pflanzenteilen‐in‐nahrungsergaenzungsmitteln.html
Belgium	As per the most recent amendment to the Royal Decree on Botanicals of August 31, 2021, AS (whole plant) remains on List 3 (mandatory product notification).	https://www.health.belgium.be/sites/default/files/uploads/fields/fpshealth_theme_file/kb_31‐08‐2021_geconsolideerd.pdf
Bulgaria	As per Foodstuff Act (2021) and Ordinance on Food Supplements (effective 2022), the placing of food supplements on the Bulgarian market must be preceded by the registration of the relevant product in the public register kept by the Bulgarian Food Safety Agency. Prohibited botanicals are listed in Annex III, Part A of Regulation (EC) No 1925/2006, listed in Annex 5 of Ordinance 5 of 2004 or classified as narcotic.	https://www.schoenherr.eu/content/bulgaria‐new‐requirements‐for‐food‐supplements/
Croatia	AS is not listed in Annex II of the Ordinance No. 160/13 of ‘other substances’ permitted for use in food supplements or in Annex III of the ordinance. In accordance with Article 9(1) of Ordinance No. 160/13, substances that are not listed in Annex II or III may only be used in food supplements if they obtain a positive decision on the inclusion of the product in the monitoring program (mandatory notification).	https://faolex.fao.org/docs/pdf/cro138211.pdf
Cyprus	There is no list of permitted or prohibited plants for use in food supplements. Food supplements containing botanical ingredients are evaluated by the National Committee on Food Supplements.	https://food.ec.europa.eu/document/download/bc1927f5‐9eba‐4ed1‐ba79‐d70c38f9cffc_en
Czech Republic	Per the decree on food supplements and food composition 58 (2018), the use of AS and extracts thereof are permitted, provided the authorities are notified.	https://faolex.fao.org/docs/pdf/cze192343.pdf
Denmark	OVERRULED. AS is included in the Danish *Drogelisten* which has been developed based on previous product evaluations of food supplements.	https://foedevarestyrelsen.dk/kost‐og‐foedevarer/alt‐om‐mad/kemi‐i‐maden/mad‐med‐uoensket‐kemi/ashwagandha
Estonia	AS is included in the Estonian State Agency of Medicine's list of substances and plants that have therapeutic properties and could be defined as a medicinal product (while still referred to in a 2020 guidance document, the list has since been removed), compiled in accordance with the Minister of Social Affairs Regulation (SAM) No. 59 of 2005. To determine if any given final product is a medicine or a food supplement, SAM classifies the product on a case‐by‐case basis.	https://pta.agri.ee/sites/default/files/documents/2020‐12/tl_hindamine_eng_sept_2020.pdf
Finland	AS is not included in the list of herbals published by the Finnish Medicines Agency (Fimea) that may qualify a product as medicinal. It could in principle be permitted for use in food supplements, provided the authorities are notified.	https://www.ruokavirasto.fi/en/foodstuffs/food‐sector/product‐and‐industry‐specific‐requirements/food‐supplements/questions‐about‐food‐supplements/
France	OVERRULED. AS is not included in the June 2014 Order on Plants authorized in food supplements. Two alternative notification procedures should therefore be followed for the marketing of a food supplement containing AS in France: either an Article 16 declaration procedure (mutual recognition) or an Article 17 declaration procedure pursuant to the French Decree on food supplements, which requires the submission of a pre‐marketing authorization dossier to the French food authorities DGCCRF and a case‐by‐case safety assessment from ANSES. In either case, the food business operator must possess a dossier on the characterization of the plant preparation (content in line with the Annex II of the French Plant Order). The French Pharmacopoeia (2014 version) includes AS root in list B: “Traditionally used medicinal plants, as such or as plant preparation, and for which the potential adverse effects are superior to the expected therapeutic benefit”.	https://www.legifrance.gouv.fr/loda/id/JORFTEXT000029254516
Germany	OVERRULED. AS root is recorded in the German List of Herbal Substances (*Stoffliste*) as a substance used in both foods and medicines. Food supplements containing botanicals must be evaluated on a case‐by‐case basis for compliance with the general legal provisions for food. The definitions of a food ingredient and food additive in German Food Law (LFGB) in §2 (3) are very complex and are always interpreted based on the recent outcomes of case law and the views of the German authorities.	https://www.bvl.bund.de/DE/Arbeitsbereiche/01_Lebensmittel/01_Aufgaben/07_Stofflisten/lm_stofflisten_node.html
Hungary	AS is included in the list of plants that are not recommended for use in food supplements by the expert panel of OÉTI (the National Institute for Food and Nutrition Science). The current Hungarian register of notified food supplements does, however, include successful notifications for products containing AS.	https://ogyei.gov.hu/dynamic/alkalmazasra_nem__javasolt_novenyek_202409.pdf
Ireland	To date, there is no Irish legislation on botanical ingredients for use in food supplements. AS could in principle be permitted for use in food supplements, provided the authorities are notified.	https://www.fsai.ie/business‐advice/food‐supplements
Italy	AS (whole plant) is included in the currently applicable Italian list of permitted plants/plant preparations for use in food supplements. The most recent (uncoupled) Italian version of the BELFRIT list is included as an annex to a decree published in the official gazette 224, 2018. The list does not include any specific notes related to required warning statements or specifications/limitations related to its active compounds for this plant.	https://www.gazzettaufficiale.it/atto/serie_generale/caricaDettaglioAtto/originario?atto.dataPubblicazioneGazzetta=2018‐09‐26&atto.codiceRedazionale=18A06095&elenco30giorni=true
Latvia	Not listed in *Regulations Regarding the Plants and Parts of Plants Prohibited for Human Consumption*, AS could in principle be permitted for use in food supplements, provided the authorities are notified.	https://likumi.lv/ta/en/en/id/320191‐regulations‐regarding‐the‐plants‐and‐parts‐of‐plants‐prohibited‐for‐human‐consumption
Lithuania	AS is included in Order No V‐432 approving the Lithuanian Hygiene Norm HN 17:2010 on Food Supplements, as amended, in the list of prohibited food supplement ingredients of plant origin. Nonetheless, three products with AS are included in the register of notified food supplements.	https://e‐seimas.lrs.lt/portal/legalAct/lt/TAD/431c6e31a65011e68987e8320e9a5185
Malta	Legislation does not specifically allow or prohibit the use of herbs in food supplements. AS could in principle be permitted for use in food supplements, provided the authorities are notified.	AS could in principle be permitted for use in food supplements, provided the authorities are notified or the product falls under mutual recognition (Regulation (EU) 2019/515)
Netherlands	OVERRULED. The current *Warenwetbesluit Kruidenpreparaten* does not list AS. AS extract could therefore in principle be used in food supplements if it is safe and no medicinal claims are made or concerns raised.	https://wetten.overheid.nl/BWBR0012174/2022‐07‐01#Bijlage
Norway	AS is indicated in the Norwegian Regulation FOR 1999‐12‐27‐1565, as amended, as a prescription medicine.	https://lovdata.no/dokument/SFO/forskrift/1999‐12‐27‐1565
Poland	OVERRULED. The use of AS in food supplements in Poland is not regulated and may be used in supplements, provided the authorities are notified.	https://www.gov.pl/web/gis/zespol‐do‐spraw‐suplementow‐diety
Portugal	Portuguese Decree No 136/2003, as amended, on food supplements permits the use of botanicals and other bioactive substances in food supplements. AS may be used in supplements, provided the authorities are notified.	https://files.diariodarepublica.pt/1s/2003/06/147a00/37243728.pdf?lang=EN
Romania	Annex A of the Romanian Order 244/401/2005 includes a list of prohibited (list 1) and a list of permitted (list 3) plants. AS is not included in either of these lists. Food supplements containing AS have been successfully notified and are listed in the database of notified food supplement products.	https://legislatie.just.ro/Public/DetaliiDocument/62073
Slovakia	The use of AS in food supplements in Slovakia is not specifically regulated. AS may be used in supplements, provided the authorities are notified.	https://www.uvzsr.sk/web/uvzen/registration‐of‐food‐supplement
Slovenia	Purportedly, both positive and negative lists existed for the use of plants in foodstuffs, which were replaced with a guidance document issued by the competent authority. While secondary references to this guidance document were identified, the document itself could not be located.	https://www.gov.si/teme/prehranska‐dopolnila/
Spain	The use of plant preparations in food supplements is not specifically regulated in Spain. Spanish authorities follow a flexible approach based on mutual recognition. AS is, however, included in a negative list restricting its use due to purported toxicological concerns, the legality of which, however, has been questioned (https://eur‐lex.europa.eu/legal‐content/EN/TXT/PDF/?uri=CELEX:62007CJ0088).	https://www.boe.es/eli/es/o/2004/01/28/sco190
Sweden	AS is not indicated in the Swedish list of plants not suitable for food use: *Förteckning över växter och växtdelar som är olämpliga I livsmedel* (VOLM). The list, however, appears to have been replaced with EU guidance. AS could therefore in principle be permitted for use in food supplements.	https://kontrollwiki.livsmedelsverket.se/artikel/78/kosttillskott
Switzerland	Harmonized with *Stoffliste* (see Germany, not clear if overruled), maintains its own negative list, however.	https://www.fedlex.admin.ch/eli/cc/2017/181/de

Additionally, as a longstanding EU membership candidate, Turkey maintains its own list of substances with use restrictions in supplements, which currently does not include an entry for AS.^2^

### 3.3 AS In the United Kingdom

Prior to discussing the position of botanicals and their legislative controls in the United Kingdom (UK) it is important to understand the country's current legal framework since its exit from the European Union. Until January 31, 2021, the UK was a member of the EU and was therefore bound by European law. Since leaving the EU, the UK transposed all legislation related to foods and drugs into national law and, as such, retains a degree of continuity with its historic rules (UK Parliament 2018). However, if a challenge to a specific piece of assimilated EU law were to take place in the UK courts, then arguments based on EU principles such as supremacy, proportionality and subsidiarity would be considered inoperable (UK Parliament 2023). These freedoms give the UK the right to develop and interpretate legislation on botanicals in a manner that differs from the EU, and indeed, on issues such as risk assessment, there has been divergence, for example, on the safety of titanium dioxide (Committee on Toxicity 2024).

In the UK, there are several routes available to introduce herbal products to the market, and the applicable legislation is based on their classification as either a food or drug. When considering the structure of botanical legislation in the UK and the legal sale of AS, traditional herbal medicines, human medicines and food laws are applicable. As it relates to a medicinal classification, the *Herbal Aide‐Memoire* compiled by the Medicines and Healthcare products Regulatory Agency (MHRA) serves as guidance document (MHRA 2005). While this document has no legal status (similar to the novel foods catalogue), it is indicative for the purpose of classification. The *Memoire* lists AS as having a history of medicinal (root and leaves) and cosmetic use, yet no history of food use. Such a classification is to be considered on a case‐by‐case basis, giving due regard to dose, consumer perception and other relevant factors as evidenced by the many AS food products sold in the UK.

Although AS has a long history of use in Ayush, there is no approval at this time under the traditional herbal medicinal products regulations (HMPC 2013). However, since Brexit, the MHRA has indicated that it may be able to accept evidence of traditional use from countries in addition to EU/EEA (MHRA 2020). To date, however, no registration has been granted for AS despite such opportunities. Although there is no approval, there is also no ban under medicines laws (Schedule 20, Regulation 241 of the Human Medicines Regulation 2012). Of note, there are substances on the banned list, for example, slippery elm, *Ulmus rubra* L., due to its traditional use as an abortifacient (pers. comm. MHRA).^3^ Thus, it is possible to restrict the sale of AS if the evidence demonstrates a similar risk.

In food legislation, botanicals are partially controlled under Regulation 1925/2006 (see above). The process to add substances to this list in the UK follows the provisions dictated in EU legislation. Substances authorized or restricted post‐Brexit can be found in the Great Britain Register on the addition of vitamins and minerals and of certain other substances to foods (GB VMS register) (Department of Health and Social Care 2021).

On July 8, 2024, the Food Standards Agency (FSA) launched a call for evidence on the use of AS in food supplements due to safety concerns raised in international risk assessments (Food Standards Agency 2024). This process forms part of the requirements to consult with any interested parties prior to modification of the GB VMS register. The consultation is restricted to the assessment of food supplements. This is unusual as food use is restricted to only non‐concentrate aqueous infusions from the roots with all other preparations consider novel in foods (EU Directorate‐General for Health and Food Safety 2024). As such, it is not clear why the FSA has excluded foods which have greater restrictions in use and, more importantly, are likely to be consumed in larger serving sizes than small unit quantities associated with food supplements. If the concern is one of both safety and novel food status it would have been logical to include traditional food and food supplement forms in the consultation.

The FSA consultation ended September 2, 2024, and the collated data will form part of the assessment carried out by the Committee on Toxicity (COT) that will inform FSA policy decisions. The final output is likely a consideration of the addition of AS to the Annex III of the GB VMS, in which case plant part, dose or other restrictions are to be determined.

### 3.4 AS In the United States of America

In the United States, herbal products such as AS are regulated by the Food and Drug Administration (FDA) based on their intended use. AS is primarily regulated by FDA as a dietary supplement, which is a subcategory of food. Some proprietary forms of AS have been independently affirmed as General Recognized as Safe (GRAS), that is, self‐affirmed as GRAS without FDA review, and therefore may be used in certain conventional food products as well as dietary supplements. AS can also be used in cosmetic products such as lotions and serums. However, it is most commonly marketed as a dietary supplement for health and wellness benefits, including stress relief and sleep support. The Federal Food, Drug, and Cosmetic Act (FD&C Act) and FDA's implementing regulations provide the regulatory frameworks for each category.

The FD&C Act, as amended by the Dietary Supplement Health and Education Act of 1994 (DSHEA), defines a “dietary supplement” as a product intended to supplement the diet that contains one or more of the following dietary ingredients: (a) a vitamin; (b) a mineral; (c) an herb or other botanical; (d) an amino acid; (d) a dietary substance for use by man to supplement the diet by increasing the total dietary intake; or (f) a concentrate, metabolite, constituent, extract or combination of any ingredient described in clause (a) through (e), Section 201(ff)(1) of the FD&C Act (FD&C Act 2023a). AS falls under both (d) and (f) but is often sold in extract form.

The FD&C Act Section 201(ff)(3)(B) also excludes from the definition of “dietary supplement” an article that was approved as a drug, licensed as a biologic or authorized for investigation as a drug unless prior to such approval, licensing or authorization, the ingredient was marketed as a dietary supplement or as a food (FD&C Act 2023a). Under this provision, often referred to as the drug exclusionary clause, FDA may issue a regulation finding that the ingredient, when used as or in a dietary supplement, would be lawful under the FD&C Act. Similar language in the FD&C Act applies to conventional food products, although it is included in Section 301 as a prohibited act rather than an exclusion from the definition of “food.” Section 301(ll) of the FD&C Act prohibits “[t]he introduction or delivery for introduction into interstate commerce of any food to which has been added a drug approved under section 505, a biological product licensed under section 351 of the Public Health Service Act, or a drug or a biological product for which substantial clinical investigations have been instituted and for which the existence of such investigations has been made public” unless certain conditions are met, for example, if the substance was marketed in food prior to such approval, licensure or authorization, or if FDA has issued a regulation approving the use of the substance in food (FD&C Act 2023b). Currently, AS has not been approved as a drug or licensed as a biologic, and it does not appear that AS has been authorized for investigation as a drug.

Even if an ingredient falls under one of the categories within the definition of “dietary supplement” and is not subject to the exclusionary clause, DSHEA (Section 413(d) of the FD&C Act) further classifies ingredients into one of two categories: (1) a pre‐DSHEA or old dietary ingredient (ODI) or (2) a new dietary ingredient (NDI). DSHEA defines an NDI as “a dietary ingredient that was not marketed in the United States before October 15, 1994” (FD&C Act 2023d). According to Section 413(a)(2) of the FD&C Act, if a dietary ingredient is an NDI, a premarket safety notification must be submitted to FDA at least 75 days before introducing the product into interstate commerce (FD&C Act 2023c). However, as per Section 413(a)(1) of the FD&C Act, if the NDI has been present in the food supply as an article used for food in a form in which the food has not been chemically altered, then it is exempt from NDI notification requirements (FD&C Act 2023d).^4^

Although not explicitly defined in DSHEA, ODIs are regarded as ingredients that were marketed as dietary supplements in the United States prior to October 15, 1994. ODIs may be used in dietary supplements without a premarket safety notification, depending on how the ingredient is manufactured. While not formally recognized by FDA, industry trade associations have compiled lists of dietary ingredients that are considered ODIs, for example, the list compiled by the United Natural Products Alliance (UNPA). AS appears on industry ODI lists, and it is generally accepted that this ingredient is an ODI that does not require a notification to FDA. However, if the AS is being manufactured in a manner that differs from its manufacturing as an ODI, this may convert the ingredient into an NDI.^5^

Dietary supplements marketed in the United States are subject to additional requirements enforced by the FDA, including Good Manufacturing Practices, certain requirements under the Food Safety Modernization Act, labeling and claims requirements, and adverse event reporting and recordkeeping. Specific adulteration (safety) standards under the FD&C Act also apply to dietary supplements. In addition to the adulteration provisions applicable to conventional foods, a dietary supplement is deemed adulterated as per Section 402(f)(1) of the FD&C Act if: (1) it is or contains a dietary ingredients that presents an unreasonable risk of illness or injury under the conditions of use recommended or suggested in the labeling or under ordinary conditions of use; (2) it is an NDI for which there is inadequate information to provide reasonable assurance that such ingredient does not present a significant or unreasonable risk of illness or injury; or (3) FDA determines that it poses an imminent hazard to public health or safety.

A Fact Sheet for Health Professionals prepared by the National Institutes of Health's Office of Dietary Supplements indicates that, based on “many” clinical trials, AS “has been well tolerated by participants for up to about 3 months of use” and that “[c]ommon side effects are mild,” with only few reports of serious side effects related to liver function (NIH 2023). It further notes that those who are pregnant, breastfeeding or who have hormone‐sensitive prostate cancer should not use AS. Although not required by FDA (unless the omission would cause the product to be adulterated), many manufacturers and distributors of AS supplements voluntarily include label warnings advising those who have a medical condition or are pregnant, breastfeeding or taking medication to consult with a healthcare professional prior to using the product or to avoid the product.

Similar regulatory frameworks exist for AS‐containing conventional foods and cosmetics, with some notable differences. For example, adverse event reporting is not required for conventional food products, and there is no premarket safety notification for cosmetic ingredients. As noted above, whether an AS product is regulated as a dietary supplement, food or cosmetic will depend on its intended use, which is typically determined by the product's labeling claims, advertising or other statements made about the product—although FDA may consider other evidence (21 C.F.R. §§201.128 and 801.4). Importantly, products promoted to prevent, cure, treat, mitigate or diagnose disease will be regulated by FDA as a drug and will be subject to all requirements applicable to drugs. In 2021, FDA issued a Warning Letter to a marketer of AS supplements citing website claims that provided evidence that the products are intended for use as a drug and therefore caused the products to be unapproved and misbranded new drugs, for example, “Affects the function of Natural Killer cells which reduces the inflammation in various parts of the body” and “AS also helps the body cope with anxiety, cancers, depression, arthritis, sleep disorder” (FDA 2021).

Separate from FDA‐related claims requirements, the Federal Trade Commission (FTC) enforces truth‐in‐advertising laws and sets standards for claim substantiation for a wide range of products, including dietary supplements, foods and cosmetics. Generally, claims about the health benefits or safety of foods, dietary supplements, drugs and other health‐related products must be supported by substantiation in the form of competent and reliable scientific evidence, which FTC defines as “tests, analyses, research or studies that (1) have been conducted and evaluated in an objective manner by experts in the relevant disease, condition or function to which the representation relates and (2) are generally accepted in the profession to yield accurate and reliable results” (FTC 2022). For herbs such as AS, FTC advises that advertisers consider issues such as whether clinical trials relied upon for substantiation used a product with the same extraction, composition and formulation as the advertised products, as any differences could impact efficacy. Further, claims based on traditional use, for example, in Ayurvedic medicine, are subject to the competent and reliable science evidence standard. If no such evidence exists, FTC's guidance explains how advertisers can avoid misleading consumers about the product's efficacy or about the scientific basis for any advertised health benefits. FTC also regulates other types of claims made in advertising or marketing, such as Made in the USA claims and environmental or “green” claims and imposes requirements for advertising in social media and product endorsements and testimonials. Notably, individual states may also have laws or regulations impacting AS products, for example, restricting the use of certain food additives or substances in packaging, but these products are primarily governed under federal frameworks.

### 3.5 AS In Canada

In Canada, botanical ingredients intended for ingestion have a few different routes to market. Botanical health products in dosage formats such as traditional medicinal herbs in tablets, capsules or tisanes are regulated as a category of non‐prescription drugs governed by the Natural Health Products Regulations, which were enacted in 2003 (Government of Canada 2024a). Natural Health Products (NHPs) encompass various materials including plant, algal, bacterial, fungal and non‐human animal materials, along with extracts, isolates, vitamins, minerals, amino acids, fatty acids and probiotics. Homeopathic and traditional medicines are explicitly included, and multiple routes of administration (e.g., topical or oral) are allowed, except for injection. The legal sale of NHPs in Canada requires pre‐market authorization in the form of a product license based on evidence of efficacy supporting self‐care health claims on the label, ensuring safety and quality. Canadian sites that manufacture, package, label and/or import these products must have a site license issued by Health Canada based on the evaluation of submitted evidence of compliance with good manufacturing practices.

AS is listed in the Natural Health Products Ingredients Database as the whole plant, extracts of its root, leaf and flower, and its homeopathic preparations (Health Canada 2024d). There is an AS Monograph that establishes both Ayurvedic and modern herbal medicine uses and the conditions of use that are pre‐approved for marketing AS NHPs in Canada (Health Canada 2024a). The finished product specifications must be established in accordance with the requirements described in the Natural and Non‐prescription Health Products Directorate (NNHPD) Quality of Natural Health Products Guide, which cites the specifications set out in the USP–NF, European Pharmacopoeia, British Pharmacopoeia, Pharmacopée Française, Pharmacopoeia Internationalis, Japanese Pharmacopoeia, and Food Chemicals Codex as authoritative (Health Canada 2015). As of November 18, 2024, AS is a medicinal ingredient in 200 NHP finished products that have an active license and have been notified by the licensee as being marketed in Canada (Health Canada 2024c).

In Canada, some botanical ingredients are permitted to be added to prepackaged foods for purposes other than nutrition, flavoring or food additives. Called supplemented foods, they are subject to specific limitations, including the food category to which the supplemental ingredient may be added; the maximum amount and the units per serving of stated size for a supplemental ingredient when added to a food belonging to the specified food category; types of health claims and their substantiation, the cautionary statements required on the label for each supplemental ingredient and the threshold level above which these statements are required; and any other conditions of use or additional labeling requirements related to the supplemental ingredient (Health Canada 2024e). Supplemented foods are subject to their own premarket regulations (Government of Canada 2024b).

Health Canada evaluated an application for AS root extract to be added to the List of Permitted Supplemental Ingredients but posted a negative decision online on August 6, 2024. The decision document stated that, “The assessment considered publicly available information and found the data insufficient to establish acceptable conditions for use as a supplemental ingredient in supplemented foods. Consequently, Health Canada is not acceding to the use of AS root extract as a supplemental ingredient, and there is no history of use as an ingredient added to food. Health Canada is prepared to accept a request to reconsider this decision under the regulations for supplemented foods. The Appendix of this letter identifies the information required to support reconsideration” (Health Canada 2024b).

Thus, Health Canada's evidence requirements and approach to risk mitigation of AS in NHPs versus in supplemented foods differs. For consumers' informed choice and risk mitigation, NHPs have a mandatory premarket evaluation of product license applications and finished product labeling requirements regarding the recommended conditions of use: purpose; dosage form; route of administration; dose and frequency per day; duration of use, if any; and its risk information, including any cautions, warnings, contra‐indications or known adverse reactions associated with its use (Government of Canada 2024a). In contrast, for supplemented foods there is an ingredient safety evaluation that considers its history of safe use as a food and likely level of consumers' exposure, excluding traditional herbal medicine use. Supplemented foods have much more limited risk mitigation through labeling of the maximum number of servings per day, consumption on the same day of other products with the same ingredient, and cautionary statements advising against use by certain population groups, such as those under 14 or 18 years of age, or pregnant or breastfeeding women, as warranted by the available scientific evidence (Government of Canada 2024b).

Canada's supplemented food regulatory framework is distinct from the novel food regulatory framework employed in many jurisdictions through which some botanical ingredients that are not typical foods find a route to market via a notification process. Novel foods, which are food products that are new or changed compared to existing foods, are generally intended for ad libitum consumption by anyone. Although they are separate premarket evaluation processes, Health Canada's negative decision on AS root extract as a supplemented food ingredient indicates that it would be unlikely that AS would receive a letter of no objection as a novel food unless sufficient new evidence were to be submitted (Health Canada 2022).

### 3.6 AS In Australia

As of November 18, 2024, there are 374 complementary medicines, similar to dietary supplements, containing AS that are commercially available for consumer purchase in Australia and listed on the Australian Register of Therapeutic Goods (ARTG) database administered by the national regulator, the Therapeutic Goods Administration (TGA) (ARTG 2024). All are included in the regulatory category of ‘Listed’ Medicines; that do not undergo individual pre‐market evaluation and market authorization. Listed medicines may select from permitted lists of lower‐risk ingredients and therapeutic indications that may include associated requirements such as warning statements or content restrictions. AS is included as *Withania somnifera* in the ‘Therapeutic Goods (Permissible Ingredients) Determination’; approval for its use was ‘grandfathered’ at the inception of the TGA regulatory scheme in 1990 due to its existing use at that time (Government of Australia 2024a). Any plant part and any extract type of AS is currently permitted in complementary medicines on the ARTG.

Evidence of scientific efficacy or traditional use is required. Accordingly, AS products in Australia are included in products designed for a range of health benefits, including stress, mild anxiety, energy, sleep and hormonal or perimenopausal benefits.

All complementary medicines entered into the ARTG must comply with a range of Australian safety and quality regulatory requirements. Uniquely for herbal medicines, finished products must be manufactured at the level of Pharmaceutical Inspection Co‐operation Scheme's (PIC/S) Guide to good manufacturing practice (GMP) for medicinal products, and manufacturers are subject to national TGA PIC/S GMP licensing requirements and ongoing inspections to ensure compliance. Each herbal batch is to be identified against a British Pharmacopoeia or United States Pharmacopoeia monograph or, if not available, current scientific literature and/or an authenticated botanical reference specimen; in a manner that is sufficient to discriminate between related species and/or potential adulterants/substitutes. Tablets and capsules must comply with standards for elemental impurities and residual solvents. Product sponsors have pharmacovigilance responsibilities including the reporting of serious adverse events to the TGA and the monitoring of local and international safety signals.

AS preparations for therapeutic use that do not require entry into the ARTG include raw materials, such as powdered herb or liquid extracts, which are sold to herbal medicine practitioners and may be extemporaneously compounded into individual medicines for individual patients.

The supply of AS preparations in Australia is not currently subject to the Therapeutic Goods Poisons Standard (Government of Australia 2024b), which classifies medicines and poisons with significant toxicity concerns into Schedules for inclusion in legislation of States and Territories to provide uniform restrictions on the supply, packaging and labeling, whether or not included in the ARTG.

AS has rarely been sold as a food product such as a powder. In 2023, Food Standards Australia New Zealand published a decision establishing that AS ( *Withania somnifera* ) root and root extract is a non‐traditional food with no tradition of use in Australia and New Zealand and is a novel food where safety has not been established (Food Standards Australia New Zealand 2024). AS therefore requires an application for a safety assessment of proposed patterns and levels of use before it can be sold as a food.

In 2020, the TGA identified concerns that AS may have abortifacient effects based on international monographs and evidence of traditional use. After public and industry stakeholder consultation, a mandatory warning statement (“If you are pregnant, or considering becoming pregnant, do not take without consulting a health professional”) was introduced (Therapeutic Goods Administration 2020). The warning does not apply to products that contain 12 g or less of equivalent dry root from extracts that are only water, ethanol or methanol based, in line with a 2015 reproductive and developmental toxicology study in rats (Prabu and Panchapakesan 2015).

In February 2024, the TGA published a safety advisory warning consumers to immediately stop taking AS and seek medical advice if they had any history of liver problems or were experiencing yellowing of the skin or eyes, dark urine, nausea, vomiting, unusual tiredness, weakness, stomach or abdominal pain, or loss of appetite (Therapeutic Goods Administration 2024). This warning was primarily triggered by 12 reports of liver problems to the TGA and the presence of international case reports. Of the Australian reports, seven were suspected to be related to AS, including four cases in which no other ingredients were likely to have contributed to the liver injury. At the present time, neither the TGA nor Complementary Medicines Australia have been able to identify a pattern related to a particular plant part or extract.

There were separate reports^6^ of gastrointestinal side effects including sudden severe nausea, vomiting and diarrhea. The reactions sometimes occurred after a single dose, with resolution of symptoms after discontinuation of the product. Investigations by Complementary Medicines Australia suggest these reactions are more likely to occur when a high dose of saponins is present.

Regulatory action by the TGA on liver concerns and the gastrointestinal concerns, such as mandatory label warning statements, are possible but have not yet been proposed.

## 4 Conclusion

AS is used in multiple types of products distributed and sold in most world markets, most often as dietary supplements, and therefore subject to a range of requirements enforced by national competent authorities covering the manufacturing, formulation, labeling, marketing and safety of such products. Positions adopted by national competent authorities in response to safety concerns and adverse event reports are as inconsistent as the concerns and reports themselves. Accordingly, national competent authorities should continue to undertake technical consultations on the issue, allowing countries like India to offer guidance based on the insights gained through the extensive use of AS in its vast population.

A concerted effort to address the safety concerns is urgently required, including but not limited to effects on human reproductive systems, thyroid hormones, the central nervous system and the immune system, as well as purported hepatotoxicity (based on case reports). Neither toxicological or clinical investigations of products using leaf material or mixtures of leaves and roots have yielded significant variation in composition, effect or adverse effect reporting (Williamson and Brendler 2026). Nevertheless, such substitution or admixture warrants further investigation.

Given the available evidence, European regulators are called upon conducting a balanced risk–benefit‐assessment that could result in stricter regulation (e.g., mandatory warnings), instead of an outright ban.

In addition, manufacturers of branded ingredients (extracts) are called upon to conduct and publish product‐specific toxicological investigations that follow OECD guidance and to disclose specifications for their ingredients that use non‐proprietary assay methods (e.g., as provided in standard pharmacopeias) and thus allow for comparative assessment of safe dosing and duration of consumption.

## Author Contributions

**T. Brendler:** conceptualization, data curation, investigation, methodology, project administration, writing – original draft, writing – review and editing. **R. Al‐Mondhiry:** writing – original draft, writing – review and editing. **L. Lang:** writing – original draft, writing – review and editing. **R. Marles:** writing – original draft, writing – review and editing. **M. Tallon:** writing – original draft, writing – review and editing. **A. Raghu:** writing – original draft, writing – review and editing.

## Funding

The authors have nothing to report.

## Conflicts of Interest

The authors declare no conflicts of interest.

## Data Availability

The data that support the findings of this study are available from the corresponding author upon reasonable request.

## Endnotes

^1^

https://www.internetconsultatie.nl/voedingssupplementen_en_kruidenpreparaten/b1.

^2^

https://www.tarimorman.gov.tr/Konu/1116/takviye_edici_gidalar_kisitli_maddeler_listesi.

^3^
The implementation of The Medicines (Retail Sale or Supply of Herbal Remedies) Order 1977 (SI 1977 No. 2130) into the Human medicines regulations (HMR) appears incorrect as the restrictions for whole or unpowdered bark only apply to *Ulmus fulva* Michx. in the HMR, yet the 1977 order also applied to *Ulmus rubra*. In reality, the former is a mere taxonomic synonym of the latter.

^4^
See also Section IV(B), FDA, Revised Draft Guidance for Industry: Dietary Supplements: New Dietary Ingredient Notifications and Related Issues (Apr. 2024), available at https://www.fda.gov/media/99538/download.

^5^
Ibid. Section IV(A).

^6^

https://daen.tga.gov.au/medicines‐search/.

## References

[ptr70151-bib-0005] ANSES . 2021. NUTRIVIGILANCE 2020–2021 Activity Report. ANSES.

[ptr70151-bib-0006] ANSES . 2024. Demande d'avis relatif aux risques liés à l'utilisation des préparations de *Whitania somnifera* (L.) Dunal dans les compléments alimentaires (saisine 2021‐SA‐0077). ANSES.

[ptr70151-bib-0007] ARTG . 2024. Listed Medicines Containing *Withania somnifera* Excluding Export Only Medicines. ARTG. https://compliance.health.gov.au/artg/.

[ptr70151-bib-0008] Balasubramani, S. P. , P. Venkatasubramanian , S. K. Kukkupuni , and B. Patwardhan . 2011. “Plant‐Based Rasayana Drugs From Ayurveda.” Chinese Journal of Integrative Medicine 17, no. 2: 88–94. 21390573 10.1007/s11655-011-0659-5

[ptr70151-bib-0009] BfR . 2024. Mitteilung 39, Ashwagandha: Schlafbeeren‐Präparate mit möglichen Gesundheitsrisiken. Bundesinstitut für Risikobewertung.

[ptr70151-bib-0011] Bureau of Indian Standards . 2022. Ashvagandha *Withania somnifera* L. Dunal Root Specifications for Use in Traditional Medicine. Vol. IS 18098. Bureau of Indian Standards.

[ptr70151-bib-0012] Committee on Toxicity . 2024. COT Statement on the Safety of Titanium Dioxide (E171) as a Food Additive. Food Standards Agency. https://cot.food.gov.uk/Introduction%20‐%20%28E171%29%20Executive%20Summary.

[ptr70151-bib-0013] Department of Health and Social Care . 2021. “Great Britain Register on the Addition of Vitamins and Minerals and of Certain Other Substances to Foods.” https://www.gov.uk/government/publications/register‐on‐adding‐vitamins‐and‐minerals‐to‐foods/great‐britain‐register‐on‐the‐addition‐of‐vitamins‐and‐minerals‐and‐of‐certain‐other‐substances‐to‐foods.

[ptr70151-bib-0014] DTU Fødevareinstituttet . 2020. “Risikovurdering af roden fra *Withania somnifera*.” https://foedevarestyrelsen.dk/Media/638245843133059907/Withania%20somnifera%20%20risikovurdering%2015%20maj%202020.pdf.

[ptr70151-bib-0015] EFSA . 2021. “On‐Hold Botanicals.” https://www.efsa.europa.eu/sites/default/files/2021‐06/questions‐on‐hold‐botanical‐claims.xlsx.

[ptr70151-bib-0016] EFSA Scientific Committee . 2009. “Guidance on Safety Assessment of Botanicals and Botanical Preparations Intended for Use as Ingredients in Food Supplements.” EFSA Journal 7, no. 9: 1249.

[ptr70151-bib-0017] EU Directorate‐General for Health and Food Safety . 2024. “ *Withania somnifera* (L.) Dunal.” Retrieved August 26, 2024, from European Commission. Publication no. https://ec.europa.eu/food/food‐feed‐portal/screen/novel‐food‐catalogue/search.

[ptr70151-bib-0018] European Commission . 2020. Commission Staff Working Document Evaluation of the Regulation (EC) No 1924/2006 on Nutrition and Health Claims Made on Foods With Regard to Nutrient Profiles and Health Claims Made on Plants and Their Reparations and of the General Regulatory Framework for Their Use in Foods. SWD(2020) 95 Final, Part 2/2.

[ptr70151-bib-0019] European Union . 2001. Directive 2001/83/EC of the European Parliament and of the Council of 6 November 2001 on the Community Code Relating to Medicinal Products for Human Use, as Amended.

[ptr70151-bib-0020] European Union . 2006a. Regulation (EC) No 1924/2006 of the European Parliament and of the Council of 20 December 2006 on Nutrition and Health Claims Made on Foods, as Amended.

[ptr70151-bib-0021] European Union . 2006b. Regulation (EC) No 1925/2006 of the European Parliament and of the Council of 20 December 2006 on the Addition of Vitamins and Minerals and of Certain Other Substances to Foods, as Amended.

[ptr70151-bib-0061] European Union . 2024. “Implementation Report on Regulation (EC) No 1924/2006 on Nutrition and Health Claims Made on Foods.” P9_TA(2024)0040. European Parliament.

[ptr70151-bib-0023] FDA . 2021. “Warning Letter to Orphic Nutrition.” https://www.fda.gov/inspections‐compliance‐enforcement‐and‐criminal‐investigations/warning‐letters/orphic‐nutrition‐612470‐04132021.

[ptr70151-bib-0065] FD&C Act . 2023a. 21 U.S.C. §321(ff).

[ptr70151-bib-0066] FD&C Act . 2023b. 21 U.S.C. §331(ll).

[ptr70151-bib-0067] FD&C Act . 2023c. 21 U.S.C. §350b(a).

[ptr70151-bib-0068] FD&C Act . 2023d. 21 U.S.C. §413a(1).

[ptr70151-bib-0024] Fødevarestyrelsen . 2024. “Spis ikke ashwagandha eller kosttilskud, der indeholder ashwagandha.” https://foedevarestyrelsen.dk/kost‐og‐foedevarer/alt‐om‐mad/kemi‐i‐maden/mad‐med‐uoensket‐kemi/ashwagandha.

[ptr70151-bib-0026] Food Standards Agency . 2024. “Call for Evidence: Ashwagandha.” https://www.food.gov.uk/news‐alerts/consultations/ashwagandha.

[ptr70151-bib-0027] Food Standards Australia New Zealand . 2024. “Record of Views Formed in Response to Inquiries.” https://www.foodstandards.gov.au/business/novel/novelrecs.

[ptr70151-bib-0028] FTC . 2022. “Health Products Guidance.” https://www.ftc.gov/system/files/ftc_gov/pdf/Health‐Products‐Compliance‐Guidance.pdf.

[ptr70151-bib-0029] Główny Inspektorat Sanitarny . 2021. “Zespół do spraw Suplementów Diety. Tabela 4: Maksymalne poziomy składników roślinnych oraz innych składników wykazujących efekt odżywczy i/lub fizjologiczny w suplementach diety.” https://www.gov.pl/web/gis/zespol‐do‐spraw‐suplementow‐diety.

[ptr70151-bib-0030] Government of Australia . 2024a. “Therapeutic Goods (Permissible Ingredients) Determination (No. 2).” https://www.tga.gov.au/about‐tga/legislation/legislation‐and‐legislative‐instruments/therapeutic‐goods‐determinations.

[ptr70151-bib-0031] Government of Australia . 2024b. “Therapeutic Goods Act (Poisons Standard).” https://www.legislation.gov.au/F2024L00095/asmade/text.

[ptr70151-bib-0032] Government of Canada . 2024a. “Natural Health Products Regulations (SOR/2003‐196).” https://laws‐lois.justice.gc.ca/eng/regulations/SOR‐2003‐196/index.html.

[ptr70151-bib-0033] Government of Canada . 2024b. “Regulations Amending the Food and Drug Regulations and the Cannabis Regulations (Supplemented Foods): SOR/2022‐169.” https://canadagazette.gc.ca/rp‐pr/p2/2022/2022‐07‐20/html/sor‐dors169‐eng.html.

[ptr70151-bib-0059] Government of India . 2016a. “The Drugs and Cosmetics Act 1940, as Amended.” https://cdsco.gov.in/opencms/export/sites/CDSCO_WEB/Pdf‐documents/acts_rules/2016DrugsandCosmeticsAct1940Rules1945.pdf.

[ptr70151-bib-0062] Government of India . 2016b. “Food Safety and Standards (Health Supplements, Nutraceuticals, Food for Special Dietary Use, Food for Special Medical Purpose, Functional Food and Novel Food) Regulations.” https://www.fssai.gov.in/upload/uploadfiles/files/Compendium_Nutra_29_09_2021.pdf.

[ptr70151-bib-0034] Government of India . 2023. “Survey on Ayush: 2022–2023 Fact Sheet. NSS 79th Round.” https://mospi.gov.in/sites/default/files/publication_reports/Fact_sheet‐Survey_on‐Ayush.pdf.

[ptr70151-bib-0035] Heads of Food Safety Agencies . 2024. “First Report of the HoA Working Group (Food Supplements).” https://www.bvl.bund.de/SharedDocs/Downloads/01_Lebensmittel/Internationales/Report_HoA_WG_FS‐de.pdf.

[ptr70151-bib-0036] Health Canada . 2015. “Quality of Natural Health Products Guide.” https://www.canada.ca/en/health‐canada/services/drugs‐health‐products/natural‐non‐prescription/legislation‐guidelines/guidance‐documents/quality‐guide.html.

[ptr70151-bib-0037] Health Canada . 2022. “Guidelines for the Safety Assessment of Novel Foods.” https://www.canada.ca/en/health‐canada/services/food‐nutrition/legislation‐guidelines/guidance‐documents/guidelines‐safety‐assessment‐novel‐foods‐2006.html#a3.1.

[ptr70151-bib-0038] Health Canada . 2024a. “Ashwagandha—Withania Somnifera Monograph.” https://webprod.hc‐sc.gc.ca/nhpid‐bdipsn/atReq?atid=ashwagandha&lang=eng.

[ptr70151-bib-0039] Health Canada . 2024b. “Decision on Ashwagandha Root Extract as a Supplemental Ingredient in Foods.” https://www.canada.ca/en/health‐canada/services/food‐nutrition/supplemented‐foods/list‐permitted‐food‐ingredients/information‐ingredients‐foods/decision‐ashwagandha‐root‐extract.html.

[ptr70151-bib-0040] Health Canada . 2024c. “Licensed Natural Health Products Database.” https://health‐products.canada.ca/lnhpd‐bdpsnh/?lang=eng.

[ptr70151-bib-0041] Health Canada . 2024d. “Natural Health Products Ingredients Database.” https://webprod.hc‐sc.gc.ca/nhpid‐bdipsn/.

[ptr70151-bib-0042] Health Canada . 2024e. “Supplemented Foods: Overview.” https://www.canada.ca/en/health‐canada/services/food‐nutrition/supplemented‐foods.html.

[ptr70151-bib-0043] HMPC . 2013. “Final Public Statement on *Withania somnifera* (L.) Dunal, Radix—First Version.” EMA/HMPC/681519/2012.

[ptr70151-bib-0044] IADSA . 2024. IADSA Newsflash.

[ptr70151-bib-0046] Klenow, S. , K. P. Latté , U. Wegewitz , et al. 2013. “Risikobewertung von Pflanzen und pflanzlichen Zubereitungen; 2. ergänzte Auflage.” BfR Wissenschaft, 12/13.

[ptr70151-bib-0064] Lim, X. Y. , and J. Barnes . 2024. “Ashwagandha.” Journal of Primary Health Care 16, no. 1: 112–114. 38546784 10.1071/HC23172

[ptr70151-bib-0047] Livsmedelsverket . 2022. “Dansk riskvärdering för ashwagandha kan användas i Sverige.” https://www.livsmedelsverket.se/foretagande‐regler‐kontroll/nyheter‐for‐livsmedelsforetag/nyheter‐for‐foretag/dansk‐riskvardering‐for‐ashwagandha‐kan‐anvandas‐i‐sverige.

[ptr70151-bib-0048] MHRA . 2005. Medicinal Products Containing Herbal Ingredients. MHRA. https://assets.publishing.service.gov.uk/government/uploads/system/uploads/attachment_data/file/395811/Medicinal_products_containing_herbal_ingredients.pdf.

[ptr70151-bib-0049] MHRA . 2020. “Guidance on New Provisions for Traditional Herbal Medicinal Products and Homeopathic Medicinal Products.” https://www.gov.uk/guidance/guidance‐on‐new‐provisions‐for‐traditional‐herbal‐medicinal‐products‐and‐homoeopathic‐medicinal‐products.

[ptr70151-bib-0050] NIH . 2023. “Fact Sheet for Health Professionals, Ashwagandha: Is It Helpful for Stress, Anxiety, or Sleep.” https://ods.od.nih.gov/factsheets/Ashwagandha‐HealthProfessional.

[ptr70151-bib-0051] Prabu, P. , and S. Panchapakesan . 2015. “Prenatal Developmental Toxicity Evaluation of *Withania somnifera* Root Extract in Wistar Rats.” Drug and Chemical Toxicology 38, no. 1: 50–56. 24649920 10.3109/01480545.2014.900073

[ptr70151-bib-0053] Rijksinstituut voor Volksgezondheid en Milieu . 2024. Risicobeoordeling Van Kruidenpreparaten Met Withania Somnifera (Ashwagandha) (2024‐0029). RIVM.

[ptr70151-bib-0054] Smith, T. , H. Bauman , and H. Resetar . 2024. “US Sales of Herbal Supplements Decline Slightly in 2022.” HerbalGram 139: 52–69.

[ptr70151-bib-0056] Therapeutic Goods Administration . 2020. “Outcomes: Changes to Permissible Ingredients—Low‐Negligible Risk.” https://www.tga.gov.au/outcomes‐changes‐permissible‐ingredients‐low‐negligible‐risk#fn12.

[ptr70151-bib-0057] Therapeutic Goods Administration . 2024. “Medicines Containing *Withania somnifera* (Withania, Ashwagandha): Safety Advisory – Potential for Gastrointestinal Symptoms and Very Rare Cases of Liver Injury.” https://www.tga.gov.au/news/safety‐alerts/medicines‐containing‐withania‐somnifera‐withania‐ashwagandha.

[ptr70151-bib-0063] UK Parliament . 2018. “European Union (Withdrawal) Act. Title 16.” legislation.gov.uk.

[ptr70151-bib-0060] UK Parliament . 2023. “Retained EU Law (Revocation and Reform) Act. Title 28.” legislation.gov.uk.

[ptr70151-bib-0058] Williamson, E. M. , and T. Brendler . 2026. “Ashwagandha: Is It Safe? Part 2: A Review of the Pre‐Clinical Evidence.” Phytotherapy Research 40, no. 8: 4792–4801. 10.1002/ptr.70090. 40968393 PMC13436223

